# Psychiatric Comorbidity and Emotional Dysregulation in Chronic Tension-Type Headache: A Case-Control Study

**DOI:** 10.3390/jcm11175090

**Published:** 2022-08-30

**Authors:** Rosalinda Romero-Godoy, Sara Raquel Romero-Godoy, Manuel Romero-Acebal, Mario Gutiérrez-Bedmar

**Affiliations:** 1 Department of Nursing and Physiotherapy, University of Balearic Islands, 07122 Palma, Spain; 2 Cognitive Affective Neuroscience Clinical Psychology Research Group, Institute of Health Science Research (IUNICS-IdISBa), ECYCS Research Group, University of Balearic Islands, 07120 Palma, Spain; 3 Neurology Department, Virgen de la Victoria University Hospital, 29010 Malaga, Spain; 4 Preventive Medicine and Public Health Department, School of Medicine, University of Málaga, 29010 Malaga, Spain; 5 Biomedical Research Institute of Malaga-IBIMA, 29010 Malaga, Spain; 6 CIBERCV Cardiovascular Diseases, Carlos III Health Institute, 28029 Madrid, Spain

**Keywords:** chronic tension-type headache, depression, anxiety, negative affect, emotion regulation, comorbidity

## Abstract

Background: Chronic tension-type headache (CTTH) is frequently associated with a psychiatric comorbidity of depression and anxiety. Most studies focus their attention on this association, and only few link CTTH with psycho-affective emotional regulation disorders. Objective: To evaluate the association of CTTH with anxiety, depression, positive and negative affectivity, and emotional management in CTTH patients with neither a previous diagnosis of psychiatric disorder nor use of psychoactive drugs or abuse of analgesics. Design: Case-control study. Methods: Validated scores for state and trait anxiety, depression, positive and negative state and trait affect, cognitive reappraisal, and expressive suppression were assessed in 40 subjects with CTTH and 40 healthy subjects. Associations between CTTH and psychological status were assessed through linear multivariate regression models. Results: CTTH was associated with higher scores for depression (Beta = 5.46, 95% CI: 1.04–9.88), state and trait anxiety (Beta = 12.77, 95% CI: 4.99–20.56 and Beta = 8.79, 95% CI: 2.29–15.30, respectively), and negative state affect (Beta = 5.26, 95% CI: 0.88–9.64). Conclusions: CTTH is directly associated with depression, anxiety, and negative affectivity signs despite the absence of a previously diagnosed psychiatric disorder or psychopharmacological intake. The recognition of these comorbid and psycho-affective disorders is essential to adapt the emotional management of these patients for better control.

## 1. Introduction

The International Association for the Study of Pain defines pain as: “An unpleasant sensory and emotional experience associated with, or resembling that associated with, actual or potential tissue damage”. This allows considering it as a singular psychophysical perception due to factors that may vary both interpersonally and individually in the same person over time and according to their physical, psychological, and social circumstances [1].

Tension-type headache, as defined by the latest revision of the International Headache Society (IHS), is a pathological disorder that fulfills the criteria of an essential pain without an organic basis or underlying structural damage [2]. It is the most common type of headache and is one of the most prevalent diseases globally, being the second in terms of global disease burden [3].

According IHS criteria, Chronic tension-type headache (CTTH) occurs with a frequency of more than 15 days a month or more than 180 days a year and persisting for more than 3 months [2]. It has been estimated that CTTH affects 2–3% of the general population [4], and it causes a significant functional limitation as well as a major impact on the quality of life [5,6,7,8,9].

CTTH is commonly associated with comorbidity of anxiety and depression [10,11,12,13,14,15]. Anxiety and depression are common neuropsychiatric disorders in our society, as well as in chronic pain pathologies [16], and their diagnostic clinical criteria are defined according to the Diagnostic and Statistical Manual of Mental Disorders (DSM V) [17]. Their prevalence in the Spanish population, according to the National Institute of Statistics (INE), are estimated to be 5.3% for depression and 5.8% for anxiety [18]. They are generally associated with emotional expression disorders and, at the same time, involve a disturbance in the processing and regulation of negative thinking material [19]; a reduction of negative thought material inhibition with less use of cognitive reappraisal and greater use of expressive suppression [7,20], as well as a greater faculty for rumination and difficulty in removing non-relevant negative thoughts from memory [21,22,23], have both been observed in subjects with high levels of depression. The rumination of negative thoughts generates a state of permanent tension that can contribute to the genesis of tension-type headaches. Thus, high levels of repetitive negative thinking have been associated not only with an emotional regulation deficit but also with the presence of tension headaches [23]. This situation may stay and become chronic, setting up a functional disturbance known as catastrophizing pain, that may persist even following the disappearance of the triggering factors [24].

The objective of our study was to evaluate the association of anxiety, depression, and positive and negative traits of affectivity and emotional management with patients with CTTH without a previous diagnosis of psychopathological disorder or consumption of psychotropic drugs or abuse of analgesics in order to consider a baseline situation without these influences, understand their conditions, and establish the most appropriate therapies for them.

## 2. Materials and Methods

### 2.1. Study Design and Participants Selection

The design of the present study was a case-control study. Forty subjects with a diagnosis of CTTH and another forty healthy controls (HC) with no headache were included. Cases were recruited from the Neurology Department of the Virgen de la Victoria University Hospital in Malaga (Spain). The CTTH diagnosis was made by a neurologist skilled in headaches, following International Classification of Headache Disorders criteria. Psychometric and socio-demographic data were collected by a clinical neuropsychologist.

Following a convenience-sampling method, controls were recruited among relatives or friends of patients who attended other departments of the same hospital for reasons other than neurological diseases. Controls were evaluated by a clinical interview with the clinical neurologist and neuropsychologist to avoid inter-observer error. Those who had any other illness or chronic disease, including any type of headache, were excluded.

The inclusion criteria for subjects with CTTH and HC were as follows: age between 20–69 years, with normal cognitive capacity for understanding and performing the neuropsychological tests as well as being informed and helped by the neuropsychologist.

Participants were excluded if they met any of the following criteria: having more than one type of headache (such as chronic tension-type headache and migraine), another chronic pain disease, chronic consumption of psychopharmacological and/or analgesic medication or taking any type of them at least 72 h prior to data collection, and clinical diagnosis or recognition of any neuropsychological disorder.

Cases in this study were incident cases since the Neurology Department of the Virgen de la Victoria University Hospital is a reference center for these pathologies, and all cases included were for the first time evaluated and diagnosed with CTTH.

The Ethics Committee of the University of Malaga approved this study (code number: S1033; date: 14 June 2010).

### 2.2. Psychological Status Measurement

The following questionnaires were used to collect psychological variables:

Beck Depression Inventory–II (BDI–II), to determine depression symptoms’ existence and severity, consisting of 21 items. The scoring scale is as follows: 0–9 (normal), 10–18 (mild depression), 19–29 (moderate depression), and 30–63 (severe depression), with an alpha coefficient of 0.87 [25,26].

State–Trait Anxiety Inventory (STAI), to evaluate anxiety as a temporary state (state anxiety) or as a personal characteristic (trait anxiety), consisting of 20 items each, with a Cronbach’s α coefficient ranging from 0.82 to 0.92. Anxiety is considered as a state with scores over 20.54 ± 10.56 for males and 23.3 ± 11.93 for females, and a trait for scores over 20.19 ± 8.89 for males and 24.99 ± 10.05 for females [27,28].

Emotion Regulation Questionnaire (ERQ), to separately assess cognitive reappraisal or regulation before an emotional experience and expressive suppression after an emotional experience. This test consists of 10 items: 6 items assess cognitive reappraisal, with a score of 4.73 ± 1.03 for men and 4.85 ± 1.0 for women and a Cronbach’s α coefficient of 0.79, and 4 items assess expressive suppression, with a score of 3.80 ± 1.22 for men and 3.15 ± 1.24 for women and a Cronbach’s α coefficient of 0.75 [29,30].

Positive and Negative Affect Schedule (PANAS), to evaluate subject’s emotional recognition of positive or negative affect, either as a trait or a state (trait or state positive affect, trait or state negative affect). It consists of 20 items each for both trait and state. A score of 30.23 ± 6.16 for males and 30.37 ± 6.08 for females denotes positive affect with a Cronbach’s α coefficient of 0.87–0.89, and a score of 20.61 ± 6.54 for males and 22.69 ± 6.83 for females denotes negative affect with a Cronbach’s α coefficient of 0.89–0.91 [31,32].

### 2.3. Covariate Assessment

During a face-to-face interview, the following variables were collected: age, sex, body mass index (BMI), background (if they were of urban or rural background), low socio-economic status (collected by asking subjects their yearly income and comparing it with the average Spanish salary), tertiary education (subjects were asked if they had completed a given level of studies), physical activity (subjects were asked whether or not they engaged in daily physical activity), smoking (subjects were asked if they had a daily smoking habit), and dietary intake of alcohol and coffee/tea (subjects were asked if they had a daily intake habit of both).

### 2.4. Statistical Analysis

Sample size was calculated based on the previously published study by Holroyd et al. [12] in which differences in mean scores between CTTH patients and controls were 4.1 (pooled standard deviation (SD) = 6.5) for BDI and 10.7 (pooled SD = 9.8) on the Trait Anxiety Scale of the STAI. To detect group differences with a significance level of 0.05 and a power of 0.80, 40 participants per group are necessary for BDI scores and only 14 participants per group for STAI scores. The final sample size was *n* = 80 (40 participants per group).

Characteristics of cases and controls were described as means and standard deviations (SDs) for continuous variables and percentages for categorical variables. Group comparisons were carried out using the Mann–Whitney test, Welch’s test, or Fisher’s exact test as appropriate.

Adjusted mean scores of psychological variables for levels of categorical socio-demographic variables were estimated and compared with analysis of variance. Associations between psychological variables and continuous socio-demographic variables were assessed through multivariate linear regression models.

To estimate the association between CTTH and psychological status, we adjusted a multivariate linear regression model for each psychological variable as the dependent variable. These linear models included the presence of CTTH as an independent variable and were adjusted by age, sex, and potential confounding variables to avoid any confusion bias. Potential confounders were included in the model when they were associated with CTTH or the dependent variable at a level of statistical significance of *p* < 0.25 [33] and without multicollinearity.

All statistical tests were two-sided and *p* values < 0.05 were considered statistically significant. All statistical analyses were conducted using Stata version 17.0 (StataCorp LLC, College Station, TX, USA).

## 3. Results

### 3.1. Participants’ Characteristics

Table 1 shows the sociodemographic characteristics of the study sample (CTTH vs. HC). We observed that patients with CTTH were older (50.6 years vs. 40.6 years; *p* < 0.001), had higher BMI (26.9 Kg/m^2^ vs. 23.0 kg/m^2^; *p* < 0.001), did less daily physical activity (17.5% vs. 52.5%; *p* = 0.002), had a lower educational level (15% vs. 70%; *p* < 0.001), and consumed less alcohol (2.5% vs. 22.5%; *p* = 0.014) and coffee or tea (30.0% vs. 60%; *p* = 0.013).

### 3.2. Psychopathological Characteristics of the Participants

**Figure 1:**
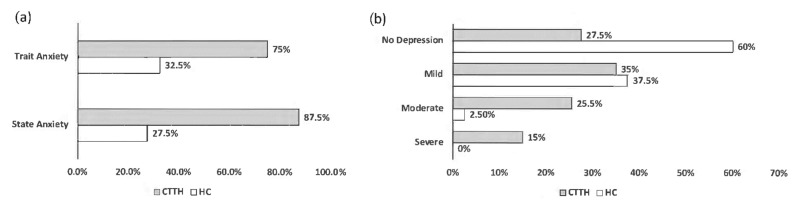
Presence of anxiety (**a**) and level of depression (**b**). CTTH: chronic tension-type headache. HC: healthy control.

We observed, employing the same psychometric inventories, depression symptoms in 40% of HC, practically all of them with mild intensity (35.5%); in the group of CTTH patients, depression symptoms were observed in 72.5%, with mild (35%) or moderate (25.5%) intensity. State anxiety symptoms were observed in 87.5% of the CTTH patients and in 27.5% of HC; trait anxiety was observed in 75% of the CTTH patients and in 32.5% of HC (Figure 1).

### 3.3. Socio-Demographic Characteristics Associated with Psychological Status

Table 2 and Table 3 show the associations between socio-demographic variables and psychological variables in the sample.

Scores for depression (Table 2) were positively associated with low socio-economic status (*p* = 0.019) and BMI (*p* = 0.007), and negatively associated with tertiary education (*p* = 0.001) and coffee or tea intake (*p* = 0.007). State anxiety scores were inversely associated with tertiary education (*p* = 0.001) and coffee or tea intake (*p* = 0.002). Low educational level and BMI were directly associated with trait anxiety scores (*p* = 0.037 and *p* = 0.002, respectively). We found higher scores for cognitive reappraisal among subjects who do physical activity (*p* = 0.001) and those with lower BMI (*p* = 0.015). Scores for expressive suppression were higher in men of older age (*p* < 0.001 and *p* = 0.001 respectively), subjects with low socio-economic status (*p* = 0.001), and subjects without tertiary education (*p* = 0.001).

Concerning affect variables (Table 3) scores for state positive affect were positively associated with tertiary education (*p* < 0.001), physical activity (*p* = 0.002) and coffee or tea intake (0.049), and negatively associated with low socio-economic status (*p* = 0.003). Trait positive affect scores were higher in subjects from urban areas (*p* = 0.034), and those who do physical activity (*p* = 0.048) and with tertiary education (*p* = 0.039). Scores for state negative affect were higher in subjects without tertiary education and daily coffee intake (*p* = 0.001 and *p* = 0.003, respectively), and with low socio-economic status and higher BMI (*p* = 0.027 and *p* = 0.018, respectively). Finally, subjects without daily coffee intake and higher BMI show higher scores for trait negative affect (*p* = 0.049 and *p* = 0.002, respectively).

### 3.4. Association between CTTH and Psychological Parameters

Table 4 shows associations between CTTH and psychological variables. It is observed that patients with CTTH are more prone to depression (regression coefficient (Beta) = 5.46, 95% Confidence Interval (95% CI): 1.04–9.88), state and trait anxiety (Beta = 12.77, 95% CI: 4.99–20.56 and Beta = 8.79, 95%CI: 2.29–15.30, respectively), and state negative affect (Beta = 5.26, 95% CI: 0.88–9.64). We observed negative associations with cognitive reappraisal and state positive affect, although only borderline significances were found (*p* = 0.098 and *p* = 0.074, respectively).

## 4. Discussion

Even though CTTH patients in our study were not previously diagnosed with depressive and/or anxiety disorders, we found a significant increase in depression and anxiety symptoms as comorbid conditions compared to HC.

According to INE sources, the incidence of depression and anxiety in the general Spanish population is 5.7 and 5.8%, respectively [18]. However, these numbers are supposedly estimated following criteria of prevalence in patients who come to the psychiatric consulting and, probably, the apparently healthy general population has a higher frequency of these psychopathologies [34].

For this reason, we preferred to use BDI–II and STAI inventories to achieve a more adequate assessment of depression and anxiety symptoms, both in CTTH patients and in HC subjects, despite the fact that a diagnosis of previous depressive and/or anxiety disorders was not present in either group. Thus, with this specific evaluation, we observed higher symptoms of depression and anxiety in both the HC and CTTH groups than expected by the INE [18] (Figure 1).

In the HC group, the prevalence of mild depression symptoms was estimated to be 40%, whereas in CTTH subjects the prevalence was 72.5%, being mild in 37.5% and moderate in 25.5%; this implies that depression symptoms appear in CTTH almost twice as frequently when compared to healthy subjects and that they are expressed with greater severity. In the HC subjects, the presence of state and trait anxiety symptoms were observed in 27.5% and 32.5%, respectively, while in CTTH subjects exhibited higher state and trait anxiety traits (87.5% and 75.5%, respectively); therefore, patients with CTTH have anxiety symptoms 2.5–3 times more frequently than healthy subjects. These findings have also been previously reported by numerous authors, most of them using psychometric assessment tests similar to those used in our study [9,11,12,13]. However, there are few references on the possible condition of dysregulation in affective and emotional expression in these patients [23] and if they do, they consider it not to be interrelated [35].

In our study we have assessed both the presence of depression and anxiety symptoms as well as affective and emotional regulation in CTTH patients without a recognized psychopathological disorder, considering that possible psycho-emotional disturbances would be causal determinants and/or influence the course of this disorder [36,37]. We observed that CTTH is associated not only with depression and anxiety, but also with a negative affect state, which implies that these subjects tend to have an emotional situation where emotions with a negative tendency predominate (such as anger, contempt, disgust, guilt, fear) [38]. This fact has also been previously appreciated, considering that high levels of negative thinking are associated with a greater emotional regulation deficit [23].

Repetitive negative thinking (whether ruminating on events that have already occurred, uncertainty, or fear of an unknown future due to excessive worry) makes people face situations with a greater state of anxiety and mood disturbance [39], reinforcing pain [40,41]. However, less negative affect conditions imply situations of greater calmness and serenity [31].

One of the main triggering and/or perpetuating factors in CTTH may be the influence of a greater negative affect that these patients have [23,42]. In our study we have found an increase in the negative state affect without a significant increase in the negative trait affect. This is a singular finding and not well-explained since it should be expected that both trait and state negative affects would be increased. This fact is not duly referenced by other authors and could be due to the characteristics of our sample, as participants might be without recognized chronic psychopathological conditions, or due to the limited number of evaluated patients.

When in confirmed psychiatric disorders, the relationship between negative affect and emotional dysregulation does not always occur, appearing in those individuals with borderline personality disorder (BPD) but not in dysthymic [43]. BPD patients have more frequent chronic headaches, and the inverse also holds [44].

A higher frequency of CTTH has been observed in patients with alexithymia (difficulty differentiating emotions) [35], however these findings could be influenced by sample characteristics, since it is not specified whether individuals in that study had a psychopathological disorder nor is it specified if they were receiving psychopharmacological or analgesic treatment that could influence emotional dysregulation [45]. It should also be considered that 55–70% of patients who come to the clinic due to headaches usually have a chronic use of medication, and most of them have an overuse or abuse [46].

We also observed that CTTH patients have a lower level of positive affective state and cognitive reappraisal. However, a larger sample would be necessary to assess whether these findings have a definitive relevance.

CTTH patients usually do symptomatic management of their symptoms with frequent consumption of psychoactive drugs due to anxiety, depression, and other psychiatric comorbidities, as well as chronic overuse of analgesics for pain [47,48] without approaching a global or multimodal physiopathological spectrum of the disease; this generates a pharmacological dependence that influences the chronification and poor control of their symptoms [49]. The use or overuse of psychoactive drugs and analgesics can alter affective states acutely during intake, during withdrawal, or as a result of chronic use [50,51].

Currently, the management of CTTH focuses especially on the symptomatic pharmacological treatment of pain, anxiety and depression comorbidity, and their repercussions (with analgesics, anxiolytics and muscle relaxants, and antidepressants); it may also be associated with other types of pharmacological and non-pharmacological options, such as: physiotherapy (electrotherapy, myofascial trigger point treatment, cervical manipulation) [52,53,54], psychological therapy (biofeedback, relaxation techniques) [55], or botulinum toxin [56], with uncertain efficacy in the medium and long terms. We believe that re-education and emotional support techniques that reinforce positive affect can contribute to a sustained supportive benefit for these patients; it has been observed that it is possible to re-educate negative thinking, and this implies better coping with pain, preventing pain chronification and catastrophizing conditions [24,57,58,59].

An important implication of our findings is the need for adding or combining psychological interventions with the management of CTTH rather than pharmacotherapy alone since a possible bidirectional relationship between CTTH and psychological comorbidities could lead to more drug dependency in these patients. Nonpharmacological therapies such as progressive muscle relaxation and deep breathing exercise have shown effectiveness in regard to pain severity, frequency, and functional status among patients with CTTH [60]. Prospective studies are needed to confirm this bidirectional relationship. This study helps in guiding a better management and treatment of CTTH, showing the importance of psychological work directed at attitude, life perspective, and the ability to face situations in a more positive and resolute way [24,57,59].

The present findings should be interpreted in the context of several limitations. First, it is possible that the small sample size may have led to no significant differences being found. Future studies with larger sample sizes and more data may support our results. Second, the neuropsychological evaluation of the CTTH patients and HC subjects was done with neuropsychological inventories and not by a psychiatric assessment, without considering other possible neuropsychiatric comorbidities in them. Third, CTTH subjects who were taking psychoactive drugs were not compared with those who were not; to assess the differences between them, it would be of interest for following studies to compare the data obtained in this analysis with other CTTH subjects with consumption of psychoactive drugs and/or analgesics overuse and assess possible differences. Finally, we have not assessed the severity of the headache and its possible relationship with neuropsychiatric symptoms.

The current study has several strengths, including that it was evaluating a special sample without previous psychopathological diagnosis, psychopharmacological treatment, or analgesic overuse or recent intake in order to consider their basal states without these determinants. The diagnosis and selection were done by a neurologist with special experience in headaches, and psychometric data were collected, face-to-face, by a trained clinical neuropsychologist. Consistent validation questionnaires in Spanish were used to assess the symptoms of depression, anxiety, affective state, and emotional management, both in the sample of CCTH and in the control group to obtain comparable results.

## 5. Conclusions

There is a high degree of association with depression and/or anxiety symptoms in CTTH subjects despite the lack of previously diagnosed psychiatric disorders or psychopharmacological intake and there is a high score of negative affectivity in them as a cause or manifestation of these disturbances. The recognition of these comorbid and psycho-affective disorders is essential to adapt the management of these patients for better control.

## Figures and Tables

**Table 1:** Characteristics of CTTH patients and healthy controls.

Characteristic	CTTH	HC	*p* Value
*n*	40	40	
Age (years)	50.6 (10.5)	40.6 (10.5)	**<0.001 ^a^**
Sex (% women)	87.5	67.5	0.059 ^c^
Smoking (%)	10.0	17.5	0.518 ^c^
Background (% urban)	75.0	82.5	0.586 ^c^
Low socio-economic status (%)	30.0	22.5	0.612 ^c^
Body Mass Index (kg/m^2^)	26.9 (4.4)	23.0 (2.2)	**<0.001 ^b^**
Physical activity (%)	17.5	52.5	**0.002 ^c^**
Tertiary education (%)	15.0	70.0	**<0.001 ^c^**
Dietary intake			
Alcohol (%)	2.5	22.5	**0.014 ^c^**
Coffee or tea (%)	30.0	60.0	**0.013 ^c^**

Data given as mean (standard deviation) or %. Statistically significant results are shown in bold (*p* < 0.05). CTTH: chronic tension-type headache. HC: healthy control. ^a^ Mann–Whitney test; ^b^ Welch’s test; ^c^ Fisher’s exact test.

**Table 2:** Adjusted ^a^ scores for Depression, State and Trait Anxiety, Cognitive Reappraisal and Expressive Suppression by socio-demographic variables.

	*Psychological Variables*									
*Socio-Demographic Variables*	Depression		State Anxiety		Trait Anxiety		Cognitive Reappraisal		Expressive Suppression	
	Adjusted Mean	*p* ^b^	Adjusted Mean	*p* ^b^	Adjusted Mean	*p* ^b^	Adjusted Mean	*p* ^b^	Adjusted Mean	*p* ^b^
Sex ^c^										
Men (*n* = 18)	12.0	0.915	23.2	0.138	24.4	0.785	4.2	0.560	4.8	**<0.001**
Women (*n* = 62)	11.8		29.4		25.3		4.4		3.3	
Background										
Urban (*n* = 17)	11.6	0.629	27.7	0.726	24.1	0.162	4.5	0.203	3.5	0.421
Rural (*n* = 63)	12.8		29.2		28.7		4.0		3.8	
Tertiary education										
Yes (*n* = 34)	7.6	**0.001**	20.8	**0.001**	21.5	**0.037**	4.5	0.708	3.1	**0.011**
No (*n* = 46)	14.9		33.3		27.7		4.3		4.0	
Low socio-economic status										
Yes (*n* = 21)	15.7	**0.019**	33.5	0.053	28.5	0.129	4.1	0.257	4.4	**0.001**
No (*n* = 59)	10.4		26.0		23.9		4.5		3.3	
Physical activity										
Yes (*n* = 28)	9.2	0.069	23.3	0.054	21.4	0.051	5.1	**0.001**	3.2	0.079
No (*n* = 52)	13.2		30.5		27.1		4.0		3.8	
Smoking										
Yes (*n* = 11)	11.9	0.980	28.2	0.967	23.7	0.672	5.0	0.132	4.0	0.312
No (*n* = 69)	11.8		28.0		25.3		4.3		3.5	
Alcohol intake										
Yes (*n* = 10)	11.3	0.841	28.3	0.631	21.0	0.252	4.4	0.982	3.6	0.914
No (*n* = 70)	11.9		25.8		25.7		4.4		3.6	
Coffee or Tea intake										
Yes (*n* = 36)	8.8	**0.007**	22.2	**0.002**	22.2	0.055	4.5	0.447	3.4	0.219
No (*n* = 44)	14.3		32.8		27.5		4.3		3.8	
Age (years)	0.129 ^d^	0.152 ^e^	−0.112 ^d^	0.464 ^e^	0.222 ^d^	0.065 ^e^	−0.007 ^d^	0.618 ^e^	0.049 ^d^	**0.001 ^e^**
Body Mass Index (Kg/m^2^)	1.089 ^f^	**<0.001 ^e^**	0.901 ^f^	0.059 ^e^	1.136 ^f^	**0.002 ^e^**	−0.108 ^f^	**0.015 ^e^**	0.086 ^f^	0.053 ^e^

^a^ Adjusted for age and sex; ^b^ F test; ^c^ Adjusted for age; ^d^ Coefficient of a linear regression model with sex as covariate; ^e^ Student’s T-test; ^f^ Coefficient of a linear regression model with age and sex as covariate. Statistically significant results are shown in bold (*p* < 0.05).

**Table 3:** Associations of socio-demographic characteristics with scores for State/Trait Positive and Negative Affect.

	*Psychological Variables*							
*Socio-Demographic Variables*	State Positive Affect		Trait Positive Affect		State Negative Affect		Trait Negative Affect	
	Adjusted Mean ^a^	*p* ^b^	Adjusted Mean ^a^	*p* ^b^	Adjusted Mean ^a^	*p* ^b^	Adjusted Mean ^a^	*p* ^b^
Sex ^c^								
Men (*n* = 18)	28.4	0.999	29.6	0.337	20.0	0.339	20.7	0.617
Women (*n* = 62)	28.4		31.6		22.2		19.8	
Background								
Urban (*n* = 17)	29.0	0.157	32.0	**0.034**	21.5	0.669	19.9	0.818
Rural (*n* = 63)	26.0		27.8		22.5		20.3	
Tertiary education								
Yes (*n*= 34)	32.4	**<0.001**	33.3	**0.039**	17.8	**0.001**	18.4	0.109
No (*n* = 46)	25.4		29.5		24.6		21.1	
Low socio-economic status								
Yes (*n* = 21)	24.0	**0.003**	29.2	0.167	25.2	**0.027**	20.5	0.644
No (*n* = 59)	29.9		31.8		20.5		19.8	
Physical activity								
Yes (*n* = 28)	32.2	**0.002**	33.4	**0.048**	19.3	0.065	19.8	0.909
No (*n* = 52)	26.3		29.9		23.1		20.0	
Smoking								
Yes (*n* = 11)	25.9	0.264	32.2	0.616	22.0	0.916	21.2	0.498
No (*n* = 69)	28.8		31.0		21.7		19.8	
Alcohol intake								
Yes (*n* = 10)	29.4	0.656	33.0	0.385	21.2	0.842	20.1	0.956
No (*n* = 70)	28.2		30.9		21.8		19.9	
Coffee or Tea intake								
Yes (*n* = 36)	30.4	**0.049**	31.4	0.798	18.6	**0.003**	18.3	**0.049**
No (*n* = 44)	26.7		30.9		24.3		21.3	
Age (years)	−0.126 ^d^	0.114 ^e^	−0.091 ^d^	0.217 ^e^	0.045 ^d^	0.592 ^e^	−0.010 ^d^	0.881 ^e^
Body Mass Index (Kg/m^2^)	−0.309 ^f^	0.215 ^e^	−0.373 ^f^	0.106 ^e^	0.619 ^f^	**0.018 ^e^**	0.626 ^f^	**0.002 ^e^**

^a^ Adjusted for age and sex; ^b^ F test; ^c^ Adjusted for age; ^d^ Coefficient of a linear regression model with sex as covariate; ^e^ Student’s *t*-test; ^f^ Coefficient of a linear regression model with age and sex as covariates. Statistically significant results are shown in bold (*p* < 0.05).

**Table 4:** Associations (multivariate analysis ^a^) between CTTH and psychological parameters.

Dependent Variable	Non-Standardized Regression Coefficient for CTTH (95% Confidence Interval)	*p*
Depression ^b^	**5.46** **(1.04, 9.88)**	**0.016**
State Anxiety ^b^	**12.77** **(4.99, 20.56)**	**0.002**
Trait Anxiety ^b,c^	**8.79** **(2.29, 15.30)**	**0.009**
Cognitive Reappraisal ^c,d^	−0.69 (−1.51, 0.13)	0.098
Expressive Suppression ^b^	0.02 (−0.77, 0.81)	0.962
State Positive Affect ^b,c^	−3.82 (−8.02, 0.37)	0.074
Trait Positive Affect ^b,c^	−2.56 (−6.82, 1.69)	0.234
State Negative Affect ^b^	**5.26** **(0.88, 9.64)**	**0.019**
Trait Negative Affect	1.90 (−1.83, 5.64)	0.312

^a^ Linear multivariate regression models adjusted by sex, age (years), tertiary education (dichotomous), body mass index (Kg/m^2^), alcohol consumption (dichotomous), and coffee or tea consumption (dichotomous); ^b^ Additionally adjusted by low socio-economic status (dichotomous); ^c^ Additionally adjusted by background (rural/urban); ^d^ Additionally adjusted by smoking (dichotomous). Statistically significant results are shown in bold (*p* < 0.05).

## Data Availability

The data presented in this study are available on request from the corresponding authors.
